# Comparing incomplete atypical femur fractures in patients with or without bisphosphonate treatment: radiography and bone morphology in a retrospective study of 19 cases

**DOI:** 10.2340/17453674.2025.43899

**Published:** 2024-06-05

**Authors:** Georg ZDOLSEK, Hans Peter BÖGL, Rickard SAND, Aneta LISZKA, Anna FAHLGREN, Jörg SCHILCHER

**Affiliations:** 1 Department of Orthopedic Surgery and Department of Biomedical and Clinical Sciences, Faculty of Health Science, Linköping University, Linköping; 2 Department of Orthopedic Surgery, Gävle Hospital, Gävle; 3 Department of Biomedical and Clinical Sciences, Division of Cell Biology, Linköping University, Linköping; 4 Center for Research and Development, Region Gävleborg/Uppsala University, Gävle; 5 Wallenberg Center for Molecular Medicine, Linköping University, Linköping, Sweden

## Abstract

**Background and purpose:**

Atypical femur fractures (AFF) are associated with bisphosphonate (BP) treatment, although 10–50% of AFF patients have never used BPs. We aimed to compare the medical history, radiographs, and bone biopsies from the fracture site of patients with AFF with (BP group) and without BP exposure (non-BP group).

**Methods:**

Between 2008 and 2021, we included 19 patients aged ≥ 50 years with incomplete AFF. During prophylactic nailing for thigh pain, a biopsy was taken that included the visible fracture line. Medical charts and radiographs were reviewed, and biopsies were analyzed histologically.

**Results:**

In the non-BP group (n = 9; mean age 70 years) patients had diseases affecting bone tissue properties (n = 3), pathological structural variations of the femur geometry or a fatigue-type mechanism (n = 3), or no identified causative patho-mechanism (n = 3). In the BP group (n = 10; mean age, 77 years) 2 patients had pathological variations of femur geometry and all used BPs. In the non-BP group, the fracture line was surrounded by bone resorption (n = 6) and cortical irregularities (n = 3), while the fracture line was restricted to a well-defined line in all patients in the BP group. The bone volume fraction (BV/TV) was on average 18% lower (95% confidence interval –35 to –1.2) in the non-BP group.

**Conclusion:**

AFF in the non-BP group are associated with bone metabolic diseases or deviations in whole-bone geometry and have a specific radiographic appearance at the fracture site whereas antiresorptive treatment appears to be the predominant etiological factor in the BP group.

Bisphosphonate (BP) treatment has shown promising results in treating elderly people who have a high risk of fragility fractures. However, acceptance of these drugs has declined because of reports of atypical femur fractures (AFF) being linked to long-term BP use. Several pathophysiological mechanisms for the development of AFF have been proposed, including: femoral geometry influencing stress/strain along the femur; changes in bone matrix composition affecting the mechanical behavior of bone tissue; and suppressed targeted remodeling, leading to the accumulation of microdamage [1]. Only some of the proposed mechanisms are unique to patients who are receiving BP treatment. As AFF also occur in patients who are not receiving BP treatment [2-5], commonly observed principles of stress fracture formation might apply, including: stress beyond the adaptive threshold of the bone tissue with normal elastic resistance (fatigue-type mechanism) [6]; defective elastic resistance due to inherited or acquired changes in bone material properties (insufficiency-type mechanism) [7]; and pathological structural variations of the femur geometry, leading to increased mechanical stress in certain areas of the femur [8] (Table 1, see Appendix).

**Table 1:** Diseases potentially associated with atypical femur fractures and their hypothesized mechanisms

Disease	Number of patients/fracture significance	Suggested mechanism	References
Hypophosphatemia	4	Hypophosphatasia is a metabolic disorder marked by low levels of alkaline phosphatase (ALP). In adults, skeletal manifestations may present as osteopenia, poorly healing stress fractures in the metatarsal bones, and pseudo-fractures that are typically found on the lateral side of the femoral shaft, which can resemble BP-associated AFFs	Nguyen et al. 2018 [27]
Pycnodysostosis	7	Impairment of osteoclast function results in osteosclerosis, abnormal bone matrix, increased bone density, and brittleness, increasing the risk of pathologic fractures in the long bones	–
Osteopetrosis	4	Failure of osteoclast-mediated bone resorption leads to increased bone mass. Although the bone density is increased, the sclerotic bone is brittle, leading to fragility fractures	–
Osteoporosispseudoglioma syndrome	1	Autosomal recessive type of juvenile osteoporosis. Loss-of-function mutation in the LRP5 gene, which regulates bone formation, leads to primary osteoblast dysfunction	–
Osteogenesis imperfecta	5	A connective tissue disorder characterized by distinct skeletal features, such as reduced bone mass, hypermineralized bone matrix, numerous fragility fractures, bone deformities, and short stature	–
X-linked osteoporosis	1	Pathogenic variants in the PLS3 gene, located on the X chromosome, have been linked to skeletal fragility in hemizygous males, whereas heterozygous females may exhibit a range of clinical phenotypes, from normal bone mineral density with no fractures to early-onset osteoporosis	–
X-linked hypophosphatemia	1	Common characteristics of this condition include low bone mineral density, rickets and/or osteomalacia, along with shortening and deformities of the lower limbs, which increase the susceptibility to fractures	–
Hypocalcemia/subclinical hypoparathyroidism	11 AFF vs 58 normal femur fractures (odds ratio 15, CI 2.9–80)	Extended periods of hypocalcemia can lead to inadequate bone formation and brittle bones that are more susceptible to fractures. As PTH stimulates bone turnover, reduced PTH levels may worsen the already suppressed bone remodeling seen in patients treated with BPs	Franceschetti et al. 2013 [28]
Tumor-induced osteomalacia	2	A rare paraneoplastic disorder. Typically, benign phos-phaturic mesenchymal tumors secrete excessive amounts of fibroblast growth factor-23 (FGF23), resulting in hypophosphatemia, muscle weakness, and an increased risk of fractures	Clegg et al. 2023 [29]
Vitamin D deficiency	20 AFF vs 152 normal femur fractures (odds ratio 3.5, CI 1.7–19)	Vitamin D deficiency-induced secondary osteomalacia may increase the risks for stress and fragility fractures	Girgis et al. 2010 [30]
Hyperprolactinemia	1	Hyperprolactinemia (in this case associated with antipsychotic medication use) suppresses the release of GnRH from the hypothalamus, which in turn causes hypogonadism that contributes to bone loss. Recent research has uncovered additional hormonal factors that may disrupt bone homeostasis, such as reduced calcitonin plasma levels in hyperprolactinemic women, or elevated parathyroid hormone-related peptide levels in the plasma of women with prolactinoma	Lee et al. 2021 [31]
Paget’s disease of bone	1	This condition is marked by an imbalance in the process of bone resorption and formation. It is thought that a defect in osteoclast function causes disorganized bone resorption resulting in inadequate bone formation, bones that are biomechanically weaker, structurally disorganized, less dense, hypervascularized, and more susceptible to fractures and deformation under stress	Berger et al. 2020 [32]
Diabetes	15 AFF vs 20 normal femur fractures. 20% (n = 3) vs. 10% (n = 2) P < 0.05	Similar to the effects of high-dose bisphosphonate treatment, extended periods of low bone turnover in type 2 diabetes can lead to elevated levels of advanced glycation end-products in bone, impairment of microdamage repair, and accumulation of bone microcracks, increasing the risk of fractures	Popp et al. 2019 [33] Joshua et al. 2014 [34]
Rheumatoid arthritis	196 AFF vs 94 normal femur fractures (crude odds ratio 11, CI 1.4–80) P = 0.02	Patients with rheumatoid arthritis generally exhibit reduced bone mineral density compared with healthy individuals. Factors that contribute to increased bone loss include age, degree of disability, low body mass index, prolonged duration of the disease, systemic inflammation associated with the condition, and extended use of gluco-corticoids	Lim et al. 2018 [35] Koh et al. 2016 [36]

AFF in patients without BP treatment are very rare (incidence 1/100,000), which makes them difficult to study [5,9]. AFF in BP-treated patients are strongly related to the decreased osteoclast activity characteristic of long-term BP treatment [10], and this reduced osteoclast activity may impact not only bone remodeling, but also the healing mechanisms for incomplete stress fractures and their appearances in radiography, computed tomography (CT), and histology [11].

We aimed to investigate the differences between AFF patients with and without BP treatment through studying the patients’ data, radiographs, and bone biopsies from the fracture site.

## Methods

### Study design

**Figure 1:**
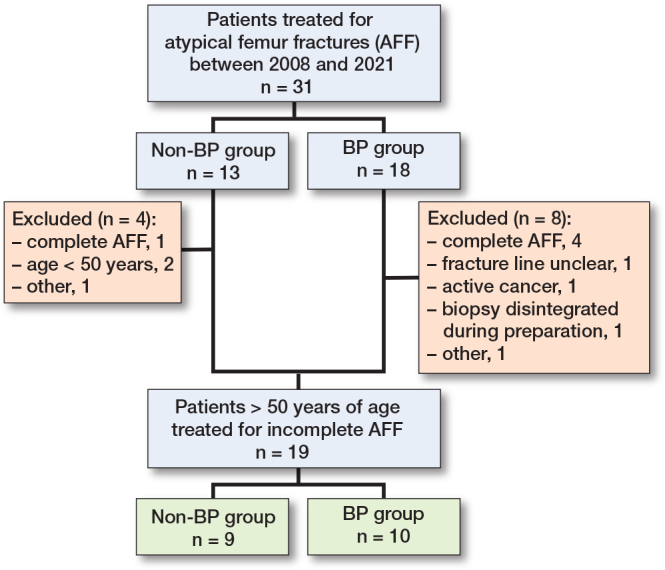
Flowchart describing recruitment to the study cohort. BP = bisphosphonate.

This retrospective study was performed at the Department of Orthopedic Surgery and the Department of Biomedical and Clinical Sciences, Faculty of Health Science, Linköping University, Sweden between 2008 and 2021. Inclusion criteria were men and women, 50 years or older with an incomplete AFF in need of surgical fixation (Figure 1).

The study is reported according to the STROBE guidelines.

### Patients: collection of radiographs and bone biopsies

**Figure 2:**
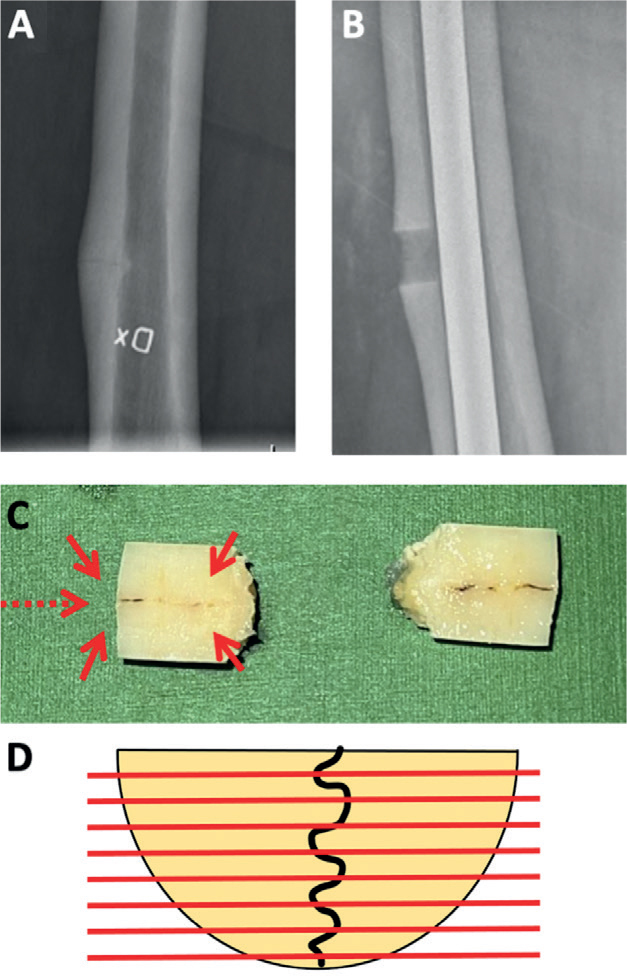
Biopsy preparation. A. Incomplete AFF. B. Cylindrical cortical defect after the biopsy. C. Cylindrical biopsies cut longitudinal with the fracture line. D. Plane of sectioning.

The patients’ characteristics, drug treatment regimens, and previous diagnoses were obtained through structured medical chart reviews. In addition, standardized patient interviews to identify diagnoses that were linked to an increased risk of stress fractures were performed before study inclusion (Table 2 and Tables S1 and S2, see Supplementary data). During the fracture fixation procedure, all the patients underwent an open bone biopsy that included the visible stress fracture lesion (Figure 2). Endocrinologic consultations were performed either before or after surgery, contingent upon the urgency of the surgical stabilization.

**Table 2:** Characteristics of the patients, fractures, and implants. Values are count unless otherwise specified

Item	Non-BP group (n = 9 ^a^)	BP group (n = 10 ^b^)
Mean age in years (SD)	70 (12)	77 (11)
Women	5	9
Treatment with BP for osteoporosis	0 ^c^	10
Treatment duration in years (SD)	0	6.7 (5.1)
range		2–21
Fracture localization		
Shaft (subtrochanteric)	6 (3)	9 (1)
Bilateral AFF	2	3
Multiple lesions	4	2
Type of treatment		
intramedullary nail ^d^	6	9
dynamic hip screw	2	0
locking plate	1	1
Potential pathologic mechanism		
Paget’s disease of bone	1	
adynamic bone disease	1	
hypophosphatemia	1	
severe coxa vara	1	
extensive lateral bowing	1	
heavy loading	1 ^e^	
unspecified	3	
bisphosphonates	–	10
extensive lateral and AP		
bowing and coxa vara	–	2

a 1 case previously published.

b 3 cases that have previously been published.

c 1 patient received a single dose of Aclasta for Paget’s disease of bone.

d All nails included the femoral head in the fixation.

e Experienced leg pain after a period of changed working conditions.

BP = bisphosphonate; AFF = atypical femur fracture;

SD = standard deviation

### Definition of AFF and radiographic analyses

**Figure 3:**
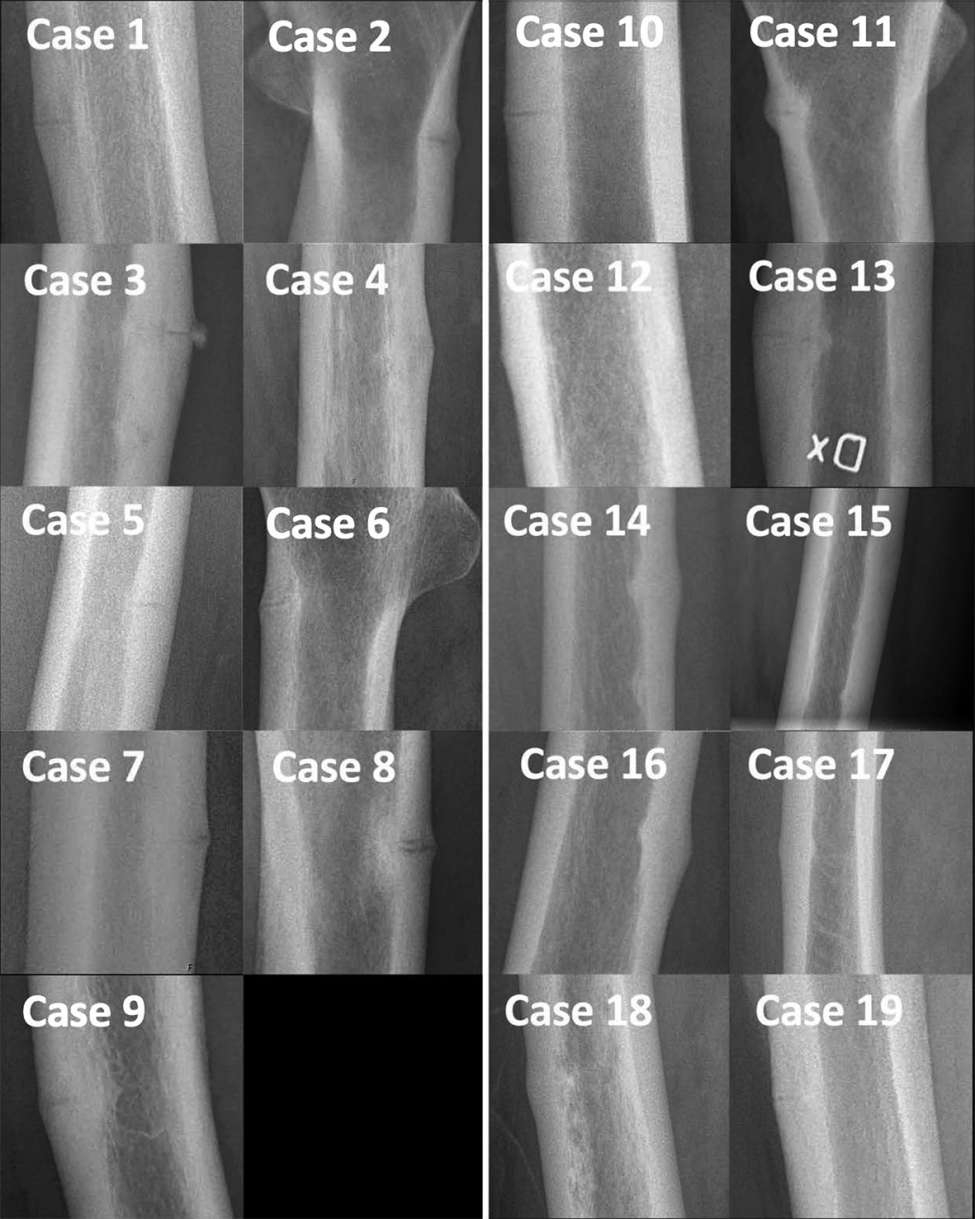
Plain radiographs with magnification at the fracture site. Note the areas of bone resorption and cortical irregularities in the patients in the non-BP group (left panel). In the BP group (right panel) the fracture line was restricted to a well-defined line or no visible fracture line on plain radiographs. BP = bisphosphonate.

AFF were defined according to the 2014 American Society for Bone and Mineral Research (ASBMR) task force major criteria. No or minimal trauma, a transverse fracture line in the lateral femoral cortex below the lesser trochanter and above the supracondylar flare, and focal cortical thickening (callus reaction) were all compulsory in our classification of AFF [12,13] (Figure 3).

Radiographic analyses were performed on standard 2-dimensional preoperative ipsilateral radiographs (Sectra IDS7 OrthoStation; Sectra AB, Linköping, Sweden). The CCD angle (neck–shaft angle) was calculated as the angle between the femoral shaft and the femoral head and neck [14]. Lateral femoral bowing was approximated on standard anteroposterior radiographs [15], and the anterior bowing angle was assessed on lateral view radiographs [16].

**Figure 4:**
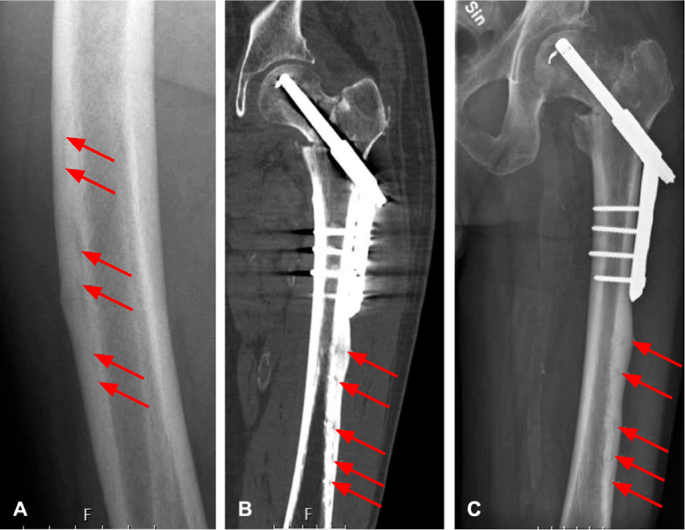
A. Cortical irregularities in proximity to mid-shaft incomplete atypical femur fracture; in Case 1. B and C. Case 3 was treated with a dynamic hip screw plate for an intertrochanteric femur fracture. On the postoperative radiograph, multiple cortical lesions are evident.

We defined subtrochanteric fractures as fractures located < 5 cm below the lesser trochanter, and diaphyseal fractures as fractures located > 5 cm below the lesser trochanter but above the supracondylar flare. The number of fracture lesions was determined on all available radiographs (Figure 4). Contralateral imaging was performed in all patients, to identify possible bilateral fractures.

### Surgery

During prophylactic fixation surgery for fractures, a biopsy that included the fracture line was taken using a cylindrical drill (diameter, 11.5 mm; Diamond TwInS; Karl Storz GmbH, Tuttlingen, Germany). Bone biopsies were fixed in 4% formaldehyde for up to 1 week and then stored in 70% alcohol. The cortical defect that resulted from the biopsy (see Figure 2) allowed the assessment of bone formation during clinical follow-up (Figure S1, see Supplementary data).

### Micro-CT

Micro-CT imaging of the bone biopsies was performed postoperatively using the SkyScan 1174 micro-CT (Bruker, Kontich, Belgium). Scans were performed at an angle of 180° with a voltage of 50 kV and using a 1-mm aluminum filter. A rotation step of 0.5°, with a frame averaging of 3 and an image pixel size of 14 μm or 16 μm was used. Reconstructions were carried out with the NRecon software (SkyScan 1174; https://www.bruker.com/en.html); beam hardening of 50% and ring artifact reduction were applied. The micro-CT data was assessed for bone volume fraction (bone tissue volume in relation to all tissue volume; BV/TV) and bone surface density (BS/TV), as well as trabecular thickness (Tb.Th), trabecular number (Tb.N.), and trabecular separation (Tb.Sp). All measurements were performed in the CT imaging software CT-Analyzer (SkyScan 1174) in regions of interest (Figure S2, see Supplementary data) and with calibrations for bone mineral density. Micro-CT images were not available in 5 cases.

### Histology

The cylindrical biopsies were cut perpendicular to the fracture line (see Figure 2) and embedded in paraffin after decalcification. 2 cases in the BP group were embedded undecalcified in poly(methyl methacrylate). Hematoxylin and eosin staining was performed for basic histologic assessment.

Bone histomorphometry and measurements of the fracture gap were performed by GZ, AL, and AF using the Olympus BX51 microscope (https://evidentscientific.com/en/search?q=Olympus+BX51+microscope+), the OsteoMeasure bone histomorphometry system (https://www.osteometrics.com/), and the cellSens Entry software (https://evidentscientific.com/en/products/software/cellsens). Using 10× magnification, an area of 100×60 μm was analyzed. The field-of-view was centered on the fracture gap and moved 60 μm (1 box) above or below the fracture gap using the tracing tool on each side of the initial box placement. The fracture gap width was measured at 5 equidistant points along the fracture line using the arbitrary line tool.

The number of osteocytes in a 750×250 μm section was counted using the 40× magnification objective. After centering on the fracture gap, the 750×250 μm grid was placed 250 μm above or below the fracture gap. Osteocyte lacunae were histologically categorized as alive if the osteocytes were present in osteocyte lacunae or were considered to be dead if the lacunae were empty. Giant osteoclasts were specifically sought [17]. GZ and AL individually assessed all the samples for the presence of loose bone fragments, woven bone close to the fracture line, cartilage adjacent to the fracture line, and osteoclasts and giant osteoclasts. These assessments were performed with the observers blinded to any background information, and consensus was reached.

### Follow-up

All patients were followed up based on individual clinical assessments. All the available medical charts and radiographs obtained during clinical fracture care were reviewed to evaluate the postoperative course of healing, including reoperations and cortical bone healing at the biopsy site.

### Statistics

The overall data were analyzed with descriptive statistics for frequencies, as percentages for categorical variables, and as means and standard deviations (SD) for continuous variables. Statistical analyses were performed using the IBM SPSS Statistics ver. 28 software (IBM Corp, Armonk, NY, USA). Methods to compensate for multiple comparisons were not applied.

### Ethics, data sharing plan, funding, use of AI, and disclosures

The study was approved by the Ethical Review Board (Dnr. M14-09, 09-03-18 and Dnr. 2011/358-31, 11-11-23), and all patients had to provide oral and written consent before study inclusion. The study was funded by ALF grants Region Ostergötland (JS), Sweden, through generous support by the Knut and Alice Wallenberg Foundation and a post-doc research grant from the Centre of Research and Development, Region Gävleborg, Sweden. AI tools were not used. The authors report no conflicts of interest. Complete disclosure of interest forms according to ICMJE are available on the article page, doi: 10.2340/17453674.2025.43899

## Results

### Patients and background characteristics

19 patients fulfilled the following inclusion criteria: 50 years or older with a need for surgical treatment for incomplete AFF (Figure 1). All of the patients underwent surgical fixation due to thigh pain that persisted for > 4 weeks and impending fracture. There were 9 patients (5 women) with no BP use (non-BP group; mean age, 70 [SD 12] years; and 10 patients (9 women) with BP use for osteoporosis (BP group; mean age 77 [SD 11] years), with a mean duration of treatment of 6.7 years (SD 5.1). The median clinical follow-up was 9 months (range 2–20) in the non-BP group and 11.5 months (range 1–57) in the BP group. The median radiographic follow-up was 15 months (range 4–31) in the non-BP group and 12 months (range 2–57) in the BP group.

**Figure 5:**
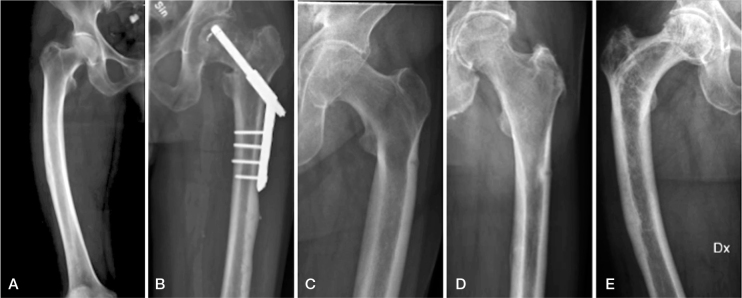
Potential pathologic mechanism in the non-BP group. A. Case 1, extensive lateral femoral bowing (CT-scout image). B. Case 3, adynamic bone disease. Note that the incomplete fractures are located below the plate fixation for the intertrochanteric fracture. C. Case 2, coxa vara. D. Case 8, hypophosphatemia. E. Case 9, Paget’s disease of bone. BP = bisphosphonate.

In the non-BP group, 3 patients had bone metabolic conditions. One patient had a clinical diagnosis of adynamic bone disease related to long-term chronic kidney disease and hemodialysis [18]. 1 patient had a diagnosis of X-linked hypophosphatemia, and 1 patient had a clinical suspicion of Paget’s disease of bone, treated with a single dose (5 mg) of zoledronic acid, 25 months before the AFF following the detection of a stress fracture lesion and signs of Paget’s disease on radiographs. As the stress fracture was already evident on MRI before the administration of zoledronic acid, this patient was assigned to the non-BP group. 2 patients had pathological structural variations of the femur geometry, 1 with coxa vara and 1 with extensive lateral bowing. Another patient suffered increasing pain after a period of intense manual labor (fatigue-type mechanism). In the remaining 3 patients, we found no clear causative factor for the AFF (Figure 5).

In the BP group, we noted some endocrinologic conditions that might affect bone health, albeit without strong associations to stress fractures (Table S2, see Supplementary data). 2 patients exhibited coxa vara and extensive lateral/anterior bowing.

### Radiography

#### Fracture characteristics

All the plain radiographs showed lesions in the lateral cortex with surrounding focal cortical thickening, representing fracture callus (see Figure 3). In 6 patients in the non-BP group, the lesions were surrounded by an area of bone resorption and in 3 patients cortical irregularities could be seen (see Figure 4). In the BP group the fracture line was restricted to a well-defined line in all the patients (see Figure 3).

Fractures located in the mid-shaft region were observed in 9 patients in the BP group and 6 patients in the non-BP group. Multiple cortical lesions were observed in 2 patients in the BP group and 4 patients in the non-BP group (see Figure 4). Bilateral AFF were found in 3 patients in the BP group and 2 patients in the non-BP group.

#### Fracture healing

Radiologic signs of healing at the biopsy site (Figure S1, see Supplementary data) were observed within 1–4 months in all the patients in the non-BP group (Table S3, see Supplementary data) and in all but 1 patient in the BP group (Table 3 and Table S4, see Supplementary data).

**Table 3:** Radiologic and histologic outcomes for the 2 groups of patients

Outcome	Non-BP group (n = 9)	BP group (n = 10)	Mean difference (CI)
Radiography			
Signs of healing within 4 months	9	9	
Healed uneventfully	7	10	
Histology and histomorphometry			
Mean width of fracture gap (SD), *μ*m	297 (191)	183 (83)	114 (–38 to 266)
Loose bone fragments	1	3	
Mainly empty osteocyte lacunae	2	8	
Osteocytes—viable (SD), %	40 (9.0)	34 (13)	5.9 (–4.7 to 17)
Woven bone close to the fracture line	9	10	
Cartilage adjacent to fracture line	0	3	
Osteoclasts	8	8	
Giant osteoclasts	0	7	
BS/TV (SD), 1/mm	45 (17)	58 (15)	–13 (–28 to 2.4)
BV/TV (SD), %	72 (7.6)	81 (5.4)	–9.3 (–16 to –2.9)

BP = bisphosphonate; CI = 95% confidence interval; BS/TV = bone surface density;

BV/TV = bone tissue volume in relation to all tissue volume.

3 patients had a complicated postoperative course despite successful fracture healing. In the non-BP group, 1 patient suffered a displaced refracture at the original fracture site 4.5 years after the index surgery (Figure S1, see Supplementary data). Another patient in the non-BP group, the patient with Paget’s disease of bone, suffered avascular necrosis of the femoral head. In the BP group, 1 patient suffered a new ipsilateral complete AFF proximal to the initial fracture, 5.7 years after the index surgery (Figure S3 and Table S4, see Supplementary data).

### Micro-CT

The mean BV/TV was 18% lower in the non-BP group than in the BP group (47% vs 65%; 95% confidence interval [CI] –35 to –1.2). All other parameters were similar between the groups (Table S5 and Figure S4, see Supplementary data).

### Histology and histomorphometry

The histologic findings confirmed those obtained in the micro-CT assessment. The mean BV/TV was 9.3% lower in the non-BP group than in the BP group (72% vs 81%; CI –16 to –2.9). All other parameters showed similar findings in both groups (Table 3 and Tables S6 and S7, see Supplementary data). Giant osteoclasts were observed only in the BP group (n = 7) (Figure S5, see Supplementary data) [19].

## Discussion

We aimed to investigate the differences between AFF patients with and without BP treatment through studying the patients’ data from medical charts, radiographs, and bone biopsies from the fracture site. We found that 6 of the AFF patients in the non-BP group had some kind of bone pathology, whereas in the BP group there were no patients with metabolic bone disorders. Similar to other studies, the patients in the non-BP group were younger and more frequently male [4,20].

### Radiography

In the assessment of plain radiographs, we found incomplete fractures in the non-BP group showing focal bone resorption around the fracture line instead of the well-defined dreaded black line in the BP group [13]. This pattern is similar to the stress lesions seen in the mid-shaft of the tibia of athletes, and is in line with current theories concerning the role of adaptive bone remodeling in the etiology of stress fractures [21,22]. Increased osteoclastic activity around the stress fracture lesion might also account for this phenomenon in the patients with AFF in the non-BP group, and it might also explain the lower BV/TV seen in the histologic analysis. These differences in radiologic fracture appearance, which have not been described previously, might impact future revisions of the ASBMR major criteria.

Extensive femoral bowing in combination with coxa vara was shown in the BP group, both of which are well-described risk factors for AFF [23]. We noted multiple cortical stress lesions in both groups [24]. This finding is rarely described in the literature, and its association with bone metabolic disease or deviating femoral geometry remains unclear.

### Micro-CT and histology

Despite differences in the pathologic mechanisms, the histologic appearance at the fracture site and the bone in close vicinity to the fracture appeared to be similar in our 2 groups, except for the findings on plain radiographs described earlier. The only finding in the histologic analysis, confirmed by similar findings from the micro-CT analysis, was an increased bone tissue volume in relation to all tissue volume (BV/TV) in the BP group. This is likely an effect of BP, as the higher mean age in the BP group would result in lower bone tissue volumes, as compared with the younger patients in the non-BP group. Another possible explanation is that the lower bone tissue volume in the non-BP group is a result of increased remodeling as part of the healing of the stress fracture lesion, as described earlier [21]. However, we found no difference in the width of the fracture gap between the groups, which means that the increased remodeling was confined to the bone around the fracture rather than at the fracture surfaces themselves.

The giant osteoclasts, which are pathognomonic for BP treatment, were seen only in patients from the BP group [19]. In a study based on 2 patients with AFF (1 with BP and 1 without BP) from Japan, the authors noted hypertrophic osteoclasts, decreased bone resorption surface, decreased osteoclast numbers on the bone resorption surface, and an increased ratio of multinuclear osteoclasts, as well as misshapen and thin osteons and a higher mass and ratio of woven bone to total bone mass in the BP-treated patient [25]. Possible explanations for the non-significant findings regarding bone microstructure in our study include a small sample size and an associated risk of a Type II error. In addition, our bone samples were only representative of the bone tissue in close vicinity to the stress fracture lesion, where the fracture healing process might occlude the effect of the BP on the bone tissue. The inhomogeneous etiology in the non-BP group might cause dispersion of our findings, thereby smoothing the differences between the groups.

### Limitations

The small sample size prohibits the making of any deductions based on statistically significant differences. Furthermore, we were restricted to the most basic investigations of bone microstructure available to us, which prohibited the analysis of BP-specific changes, such as microcracks [26]. We included only patients with symptomatic AFF necessitating prophylactic surgical fixation in both groups, which may have resulted in selection bias towards more severe cases. We also chose to include patients with underlying bone metabolic disease. However, no patients with these conditions were identified in the BP group. We believe that this approach yields a more representative sample of AFF patients in the clinical setting, where monogenic bone disorders, when looked for, appear to be relatively common [2,3].

### Conclusion

We found small clinical differences between the BP and non-BP group related to bone metabolic diseases, deviations in whole bone geometry, and the radiologic appearance at the fracture site.

*In perspective,* a femoral stress fracture in the BP-naive patient should alarm the clinician to search for possible explanations including but not limited to bone metabolic diseases or deviations in whole-bone geometry.

### Supplementary data

Supplementary Figures (S1–S5) and Supplementary Tables (S1–S7) are available in separate files on the article page, doi: 10.2340/17453674.2025.43899

## Supplementary Material





## Notes

Study design: JS, GZ, AF, data collection: GZ, JS, AL, data analysis and interpretation: JS, GZ, HPB, manuscript preparation: all authors.

Handling co-editor: Taco Gosens

*Acta* thanks Hannu T Aro and other anonymous reviewers for help with peer review of this manuscript.
