# Supplementary material for: Comparing incomplete atypical femur fractures in patients with or without bisphosphonate treatment: radiography and bone morphology in a retrospective study of 19 cases
Source: Acta Orthop. 2024 Jun 5;96:421–8. doi: 10.2340/17453674.2025.43899 (PMC12138533; doi:10.2340/17453674.2025.43899)
Supplement: Supplementary file 1 [file ActaO-96-43899-s1.pdf]

Supplementary Figures

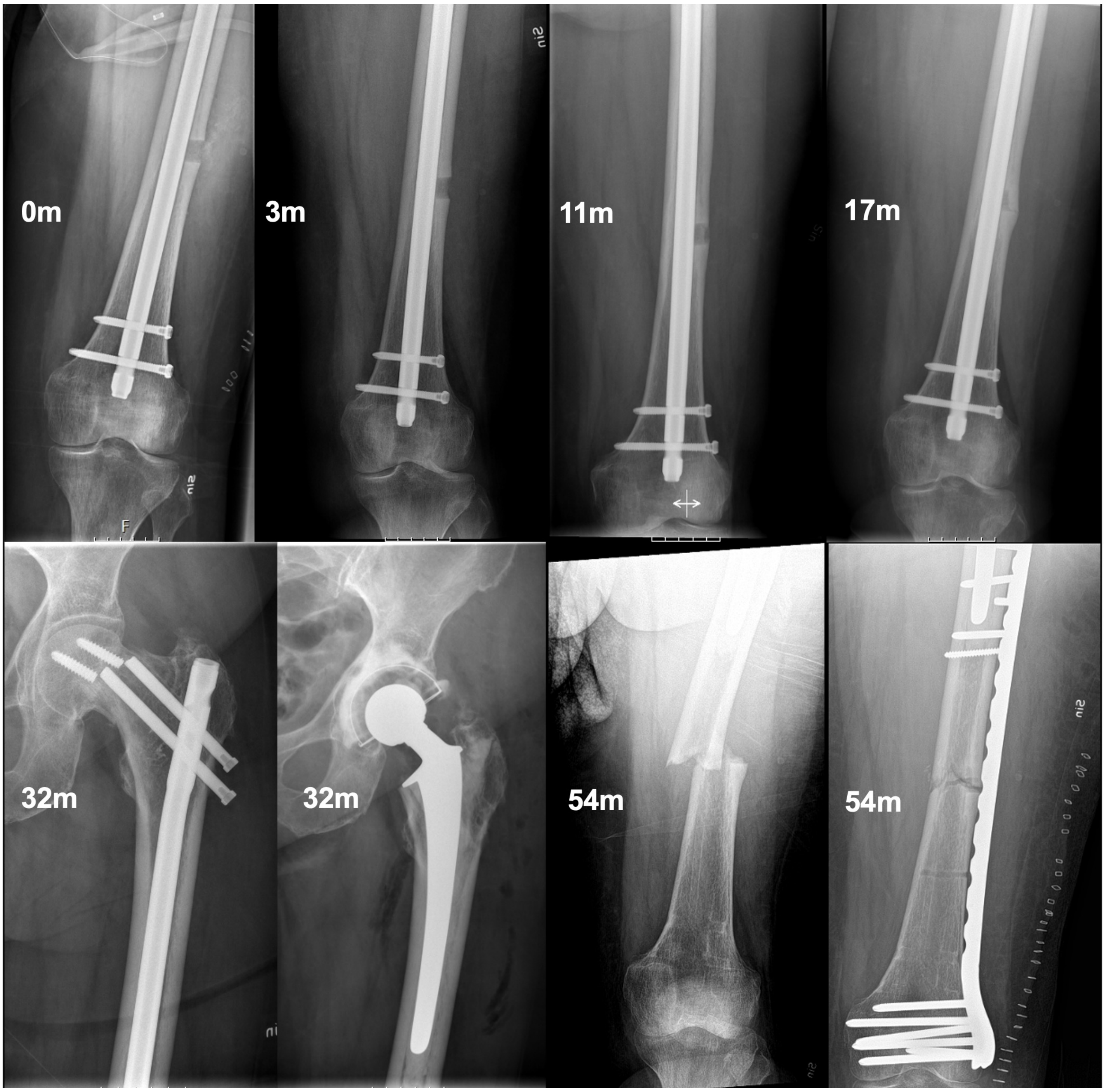

Figure S1. Patient who sustained a refracture at the original fracture site 4.5 years after index surgery and who underwent removal of the intramedullary nail 23 months previously due to a femoral neck fracture (Case 4).

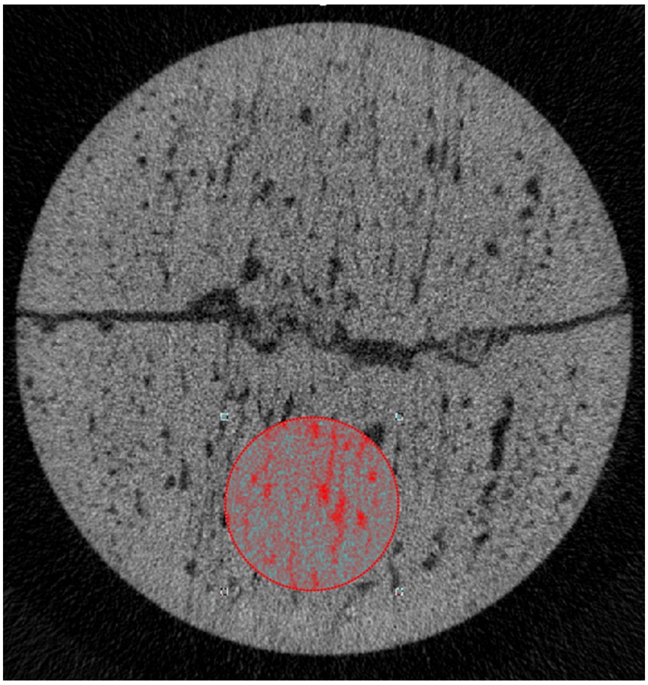

Figure S2. A region of interest (red circle, 3-mm diameter) was used to perform measurements in the CT-Analyzer (Sky-Scan 1174 micro-CT). The region of interest was chosen so as to avoid the fracture gap and sample margin, while maintaining the largest possible size throughout the samples.

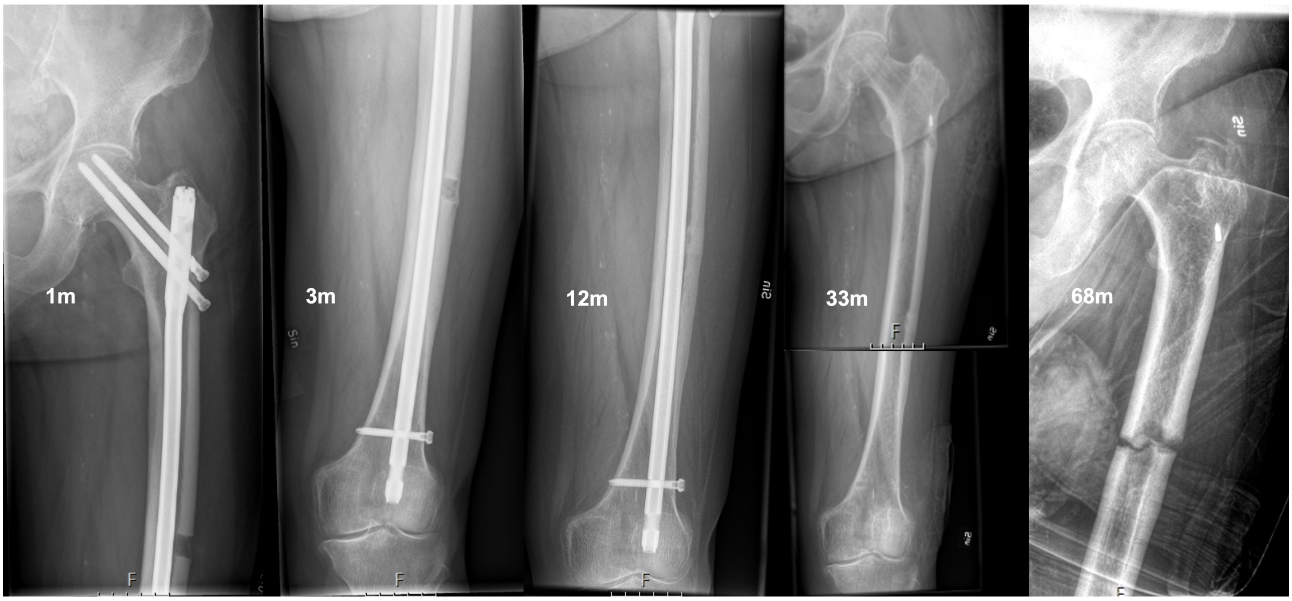

Figure S3. Patient in the *BP group* who sustained a new complete AFF proximal to the initial fracture, 5 years and 8 months after the index surgery after removal of the intramedullary nail at 33 months postoperatively owing to local discomfort at the site of nail insertion (**Case 15**).

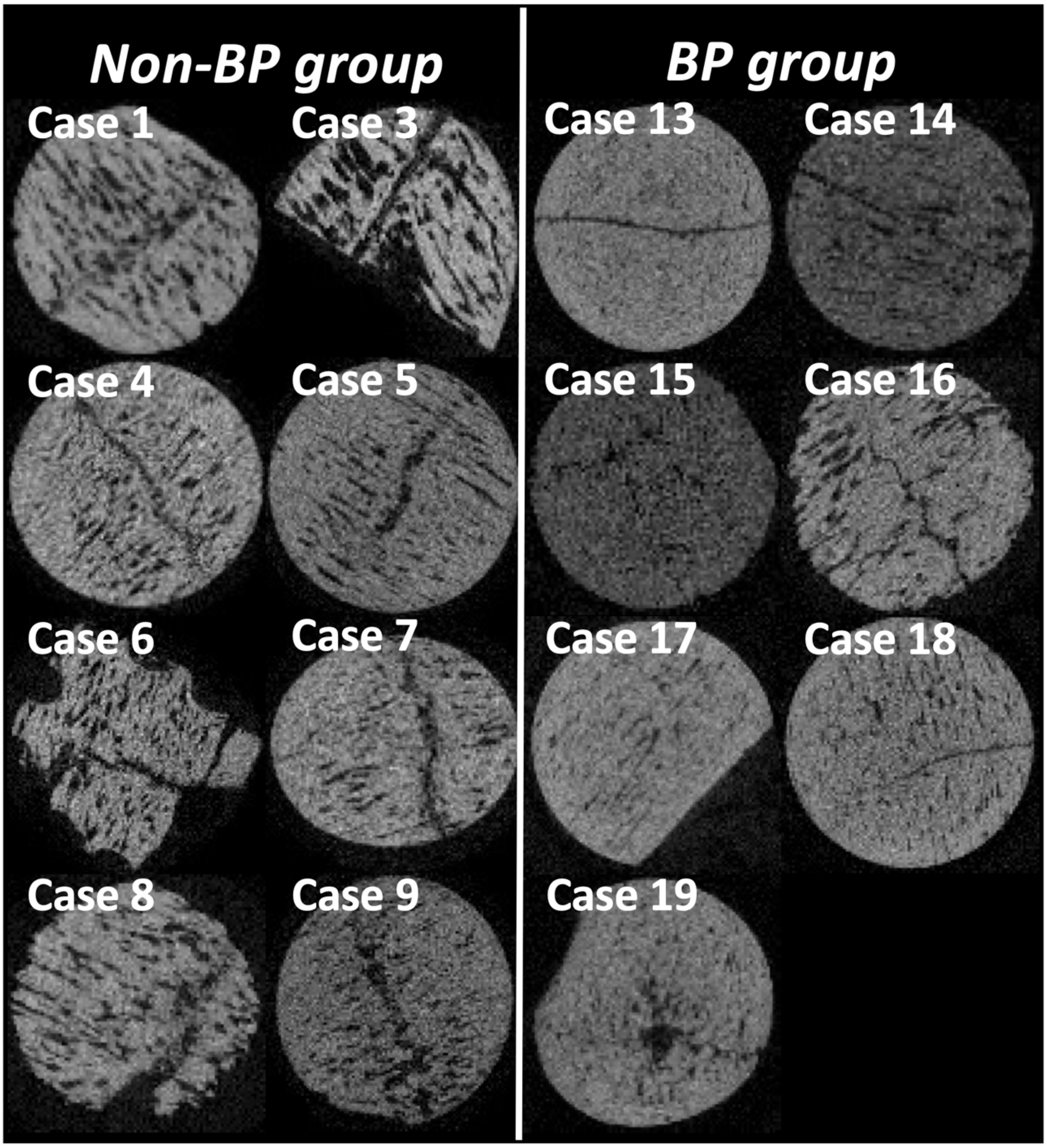

Figure S4. Representative micro-CT images of the fracture gaps in the *BP group* and *non-BP group*.

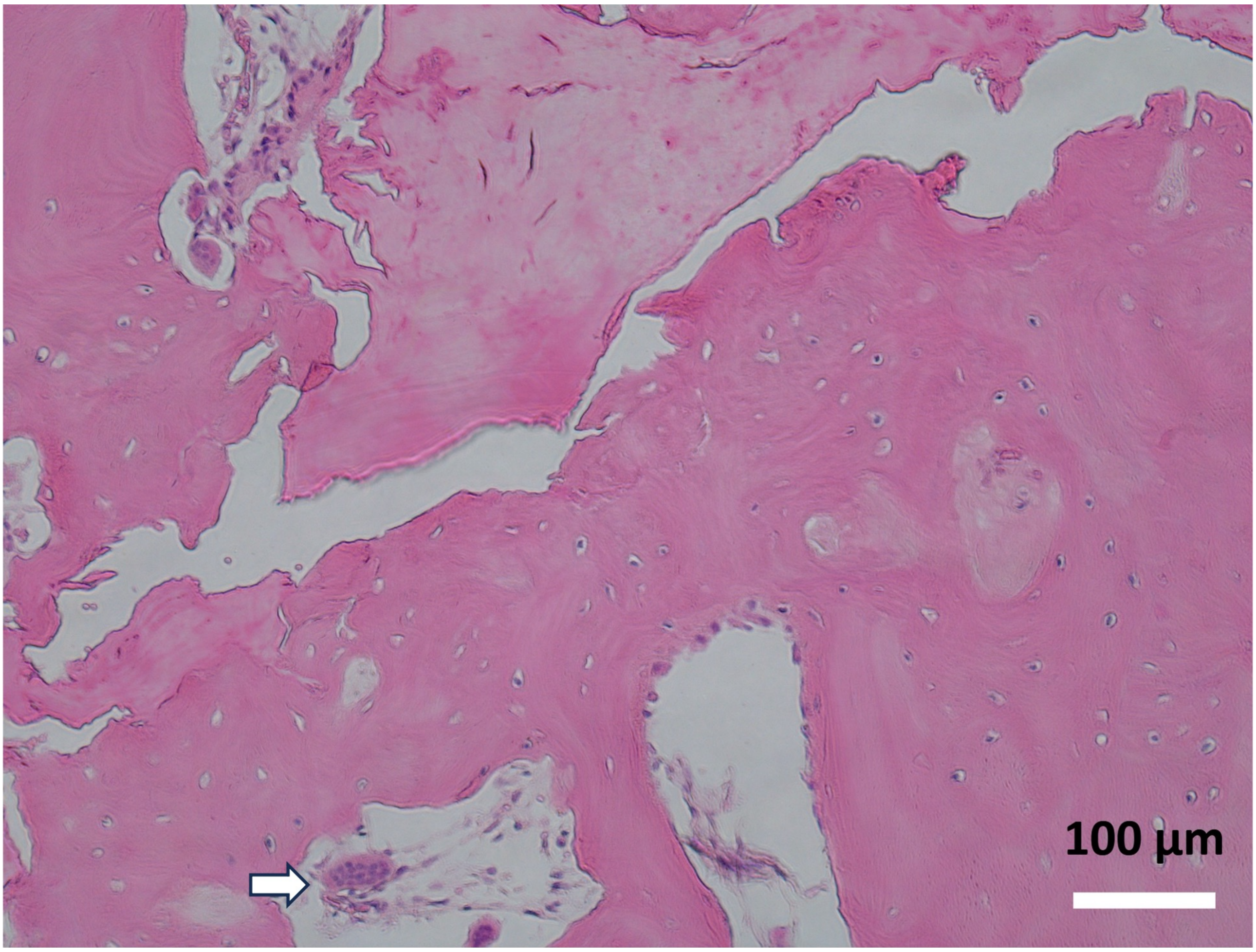

Figure S5. Representative image of an osteoclastic giant cell (arrow) in the *BP group* (Case 11) stained with hematoxylin and eosin, visualized using the 10×/0.45 M27 objective lens (Carl Zeiss).
