# Supplementary material for: Comparing incomplete atypical femur fractures in patients with or without bisphosphonate treatment: radiography and bone morphology in a retrospective study of 19 cases
Source: Acta Orthop. 2024 Jun 5;96:421–8. doi: 10.2340/17453674.2025.43899 (PMC12138533; doi:10.2340/17453674.2025.43899)
Supplement: Supplementary file 2 [file ActaO-96-43899-s2.pdf]

Table S1. Detailed information on the patients with AFF in the non-BP group

	Case 1	Case 2 [a]	Case 3 [b]	Case 4	Case 5	Case 6	Case 7	Case 8	Case 9
**Age**	83	78	64	60	62	84	63	50	82
**Sex**	Female	Male	Male	Female	Female	Female	Male	Male	Female
**Bisphosphonate treatment**	0	0	0	0	0	0	0	0	Single dose of Zoledronic acid
**Indication**	N/A	N/A	N/A	N/A	N/A	N/A	N/A	N/A	Suspected Paget's disease of bone
**Treatment duration, years**	0	0	0	0	0	0	0	0	Single infusion 25 months prior to surgery
**Relevant comorbidities**	NA	Diabetes mellitus<br>Vitamin D deficiency<br>Secondary hyperparathyroidism<br>Vasculitis	AMI<br>CKD stage 5<br>Prostate cancer<br>Anemia<br>CHF	Osteoporosis	Hypothyroidism<br>Breast cancer	Rectal cancer	Dilated cardio-myopathy	X-linked hypo-phosphatemia	Paget's disease of the bone
**Fracture location**	Mid shaft	Subtrochanteric	Mid-shaft	Mid-shaft	Mid-shaft	Subtrochanteric	Mid-shaft	Subtrochanteric	Mid-shaft
**Bilateral fractures**	No	No	No	No	Yes	Yes	No	No	No
**Bone resorption around fracture line**	Yes	Yes	No	Yes	No	Yes	No	Yes	Yes
**Multiple lesions**	No	No	Yes	No	Yes	Yes	No	No	Yes
**Number of lesions**	1	1	>1 (5)	1	>1 (2)	>1 (2)	1	1	>1 (4)
**Displacement**	No	No	No	No	No	No	No	No	No

[a] Previously published case.
[b] Died within 1 year of the index surgery.
AMI = acute myocardial infarction; CKD = chronic kidney disease; CHF = congestive heart failure.

Table S2. Detailed information for patients in the BP group with AFF

	Case 10 [a]	Case 11 [a]	Case 12 [a]	Case 13	Case 14	Case 15	Case 16	Case 17	Case 18	Case 19
**Age**	80	57	81	92	81	65	76	79	90	73
**Sex**	Female	Female	Female	Female	Female	Female	Female	Female	Male	Female
**Bisphosphonate**	Alendronic acid	Alendronic acid/ risedronic acid	Alendronic acid	Alendronic acid	Alendronic acid	Alendronic acid	Alendronic acid/ zoledronic acid	Alendronic acid	Alendronic acid	Alendronic acid
**Indication**	Corticoid treatment	Corticoid treatment	Osteoporotic fractures	Osteoporosis	Osteoporosis	Osteoporosis	Osteoporosis	Osteoporosis	Osteoporotic fracture	Osteoporosis
**Treatment duration, years**	5	6	21	5	9	5	7	2	3	4
**Discontinuation of bisphosphonate treatment**	At the time of index surgery	At the time of index surgery	1.5 years prior to index surgery	At the time of index surgery	2 years prior to index surgery	5 months prior to index surgery	2 months prior to index surgery	At the time of index surgery	At the time of index surgery	At the time of index surgery
**Relevant comorbidities**	Graves' disease<br>Diabetes mellitus<br>PMR	Rheumatoid arthritis	Bullous pemphigoid	Congestive heart failure<br>Ischemic heart disease	Hypo-thyroidism<br>Smoker	Smoker<br>Diabetes mellitus	No relevant comorbidities	Hypertension<br>Hyper-lipidemia	Hypertension<br>BPH<br>Glaucoma	Postoperatively diagnosed with hypopara-thyroidism
**Fracture location**	Mid-shaft	Subtrochanteric	Mid-shaft	Mid-shaft	Mid-shaft	Mid-shaft	Mid-shaft (bilaterally)	Mid-shaft	Mid-shaft	Mid-shaft
**Bilateral fractures**	No	Yes	No	No	Yes	Yes	No	No	No	No
**Bone resorption around fracture line**	No	No	No	No	No	No	No	No	No	No
**Multiple lesions**	No	No	No	No	Yes	Yes	No	No	No	No
**Number of lesions**	1	1	1	1	>1 (3)	>1 (4)	1	1	1	1
**Displacement**	No	No	No	No	No	No	No	No	No	No

[a] Previously published case.
PMR = polymyalgia rheumatica; BPH = benign prostatic hyperplasia.

Table S3. Radiologic follow-up for the non-BP group

	General assessment	0–2 months	3–4 months	5–8 months	12–15 months	>15 months
**Case 1**	Cortical irregularities<br>Extensive lateral femoral bowing<br>Healed uneventfully	N/A	Faint callus	Bridging callus	Continuity of cortical bone	N/A
**Case 2**	Cortical irregularities<br>Coxa vara<br>Healed uneventfully	N/A	N/A	Bridging callus	N/A	M17: Continuity of cortical bone
**Case 3**	Cortical irregularities<br>Healed uneventfully	N/A	N/A	N/A	Proximal biopsy shows continuity of cortical bone, biopsy 2 shows incomplete cortical continuity	N/A
**Case 4**	Biopsy hole did not heal completely; resulted in complete AFF 4.5 years after index surgery.	N/A	Discrete callus	N/A	Bridging callus	M31: Radiolucent line at the fracture site
**Case 5**	Fracture crack appeared to originate endosteally.<br>2–3 lesions with extensive edema on contralateral femur.<br>Healed uneventfully	No callus	Bridging callus	N/A	N/A	M20: Continuity of cortical bone
**Case 6**	Healed uneventfully	Faint callus	N/A	N/A	Continuity of cortical bone	N/A
**Case 7**	Healed uneventfully	N/A	Continuity of cortical bone	N/A	N/A	N/A
**Case 8**	Healed uneventfully	N/A	Bridging callus	Incomplete cortical continuity	Continuity of cortical bone	N/A
**Case 9**	Multiple stress lesions in Paget's disease-transformed bone<br>Healed uneventfully	Bridging callus	N/A	N/A	Continuity of cortical bone	N/A

Table S4. Radiologic follow-up for the BP group

	General assessment	0–2 months	3–4 months	5–8 months	12–15 months	>15 months
**Case 10**	Healed uneventfully	N/A	N/A	N/A	N/A	M39: Continuity of cortical bone
**Case 11**	Healed uneventfully	Faint callus	Bridging callus	Continuity of cortical bone	N/A	N/A
**Case 12**	Healed uneventfully	N/A	Faint callus	Continuity of cortical bone	N/A	
**Case 13**	Healed uneventfully	N/A	Discrete callus	Bridging callus	N/A	M26: Continuity of cortical bone
**Case 14**	Healed uneventfully	N/A	No callus	No callus	Bridging callus	N/A
**Case 15**	Extensive anterior and lateral bow<br>Coxa vara<br>Healed uneventfully;<br>Later sustained complete AFF on the same side proximal to the previous fracture level	Faint callus	Faint callus	N/A	Complete continuity of cortical bone	N/A
**Case 16**	Bilateral incomplete AFF.<br>Healed uneventfully	N/A	Right: discrete callus.<br>Left: discrete callus.	N/A	N/A	N/A
**Case 17**	Extensive anterior and lateral bow<br>Coxa vara<br>Healed uneventfully	Faint callus	Bridging callus	N/A	Completely healed	N/A
**Case 18**	Healed uneventfully	Faint callus	Bridging callus	N/A	Bridging callus	N/A
**Case 19**	Healed uneventfully	N/A	N/A	N/A	Continuity of cortical bone	N/A

Table S5. Micro CT-results for the 2 patient groups. Values are mean (SD)

**Micro CT**	**non-BP group (n = 7)**	**BP group (n = 7)**	**Mean difference (CI)**
Percent bone volume/Bone volume fraction, BV/TV (%)	47 (17)	65 (11)	–18 (–35 to –1.2)
Bone surface density, BS/TV (1/mm)	35 (7.8)	29 (5.7)	5.7 (–2.2 to 14)
Bone surface:volume ratio, BS/BV (1/mm)	80 (37)	45 (9.0)	35 (0.9 to 70)
Trabecular thickness, Tb.Th (mm)	0.05 (0.02)	0.06 (0.01)	–0.02 (–0.03 to 0)
Trabecular number, Tb.N (1/mm)	11 (2.2)	11 (2.7)	–0.03 (–2.9 to 2.8)
Trabecular separation, Tb.Sp (mm)	0.09 (0.04)	0.07 (0.03)	0.02 (–0.02 to 0.06)

Table S6. Results of the histologic analyses for the non-BP group

Histologic feature	Case 1	Case 2	Case 3	Case 4	Case 5	Case 6	Case 7	Case 8	Case 9
**Mean width of the fracture gap, µm**	143	124	587	260	123	502	250	535	149
**Loose bone fragments**	No	No	Yes	No	No	No	No	No	No
**Mainly empty osteocyte lacunae**	Yes	No	No	No	No	No	No	Yes	No
**Osteocytes – viable, %**	32	47	57	46	42	39	38	29	30
**Woven bone close to the fracture line**	Yes	Yes	Yes	Yes	Yes	Yes	Yes	Yes	Yes
**Cartilage adjacent to fracture line**	No	No	No	No	No	No	No	No	No
**Osteoclasts**	Yes	Yes	No	Yes	Yes	Yes	Yes	Yes	Yes
**Giant osteoclasts**	No	No	No	No	No	No	No	No	No

Table S7. Results of the histologic analyses for the BP group

Histologic feature	Case 10	Case 11	Case 12	Case 13	Case 14	Case 15	Case 16	Case 17	Case 18	Case 19
**Mean width of the fracture gap, μm**	154	119	145	202	187	257	235	111	353	66
**Loose bone fragments**	Yes	No	Yes	No	No	No	Yes	No	No	No
**Mainly empty osteocyte lacunae**	No	Yes	No	Yes	Yes	Yes	Yes	Yes	Yes	Yes
**Osteocytes – viable, %**	54	34	43	42	36	14	28	31	16	42
**Woven bone close to the fracture line**	Yes	Yes	Yes	Yes	Yes	Yes	Yes	Yes	Yes	Yes
**Cartilage adjacent to fracture line**	Yes	Yes	No	No	No	Yes	No	No	No	No
**Osteoclasts**	Yes	Yes	Yes	Yes	No	Yes	No	Yes	Yes	Yes
**Giant osteoclasts**	Yes	Yes	Yes	Yes	No	Yes	No	Yes	No	Yes
